# Supplementary material for: Telluriding monolayer MoS2 and WS2 via alkali metal scooter
Source: Nat Commun. 2017 Dec 18;8:2163. doi: 10.1038/s41467-017-02238-0 (PMC5735184; doi:10.1038/s41467-017-02238-0)
Supplement: Supplementary file 1 — Supplementary Information [file 41467_2017_2238_MOESM1_ESM.pdf]

Supplementary Figures

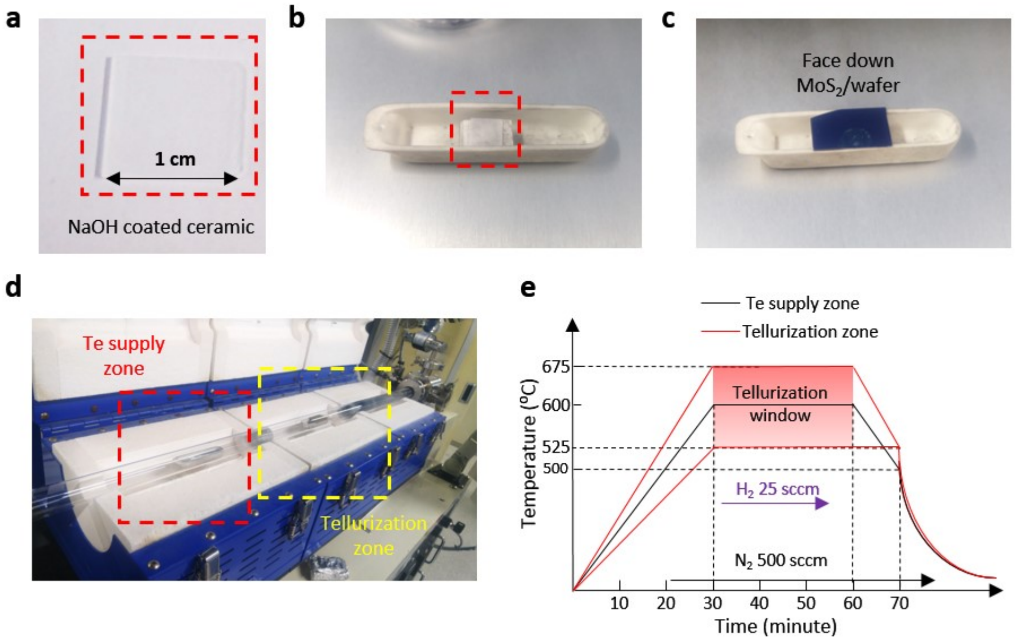

Supplementary Figure 1 | Experimental setup and temperature profile of tellurization process. a, Sodium hydroxide (0.1 g) was dissolved in 60 mL of deionized water. Of this NaOH solution, 25-100 μL was dropped onto a sapphire substrate and dried in an oven. **b,c,** The NaOH-coated substrate placed in a ceramic crucible (L 1.5 × W 5 × H 1.5 cm^3). The distance between the MoS_2 and NaOH-coated substrate can vary the feeding rate of the sodium metal, which is one of the key factors for the tellurization process. In this experiment, the distance was fixed at 2 mm by placing the NaOH substrate 1 cm above the supporter, in which the MoS_2 surface faced the NaOH substrate directly. This is also related to NaOH content. **d,** 1-inch two-zone CVD was introduced for controlling the temperature of the tellurium and conversion zones independently. **e,** The temperature profile of the tellurization process. The tellurization process was conducted at atmospheric pressure with a flow rate of 500 sccm for N_2 and 25 sccm for H_2 gas. It should be noted that H_2 gas was introduced to suppress oxidation by oxygen leakage and enhance the detachment of sulfur efficiently by hydrodesulfurization. Both zones are slowly cooled down without opening the furnace to minimize the strain between host materials and substrate.

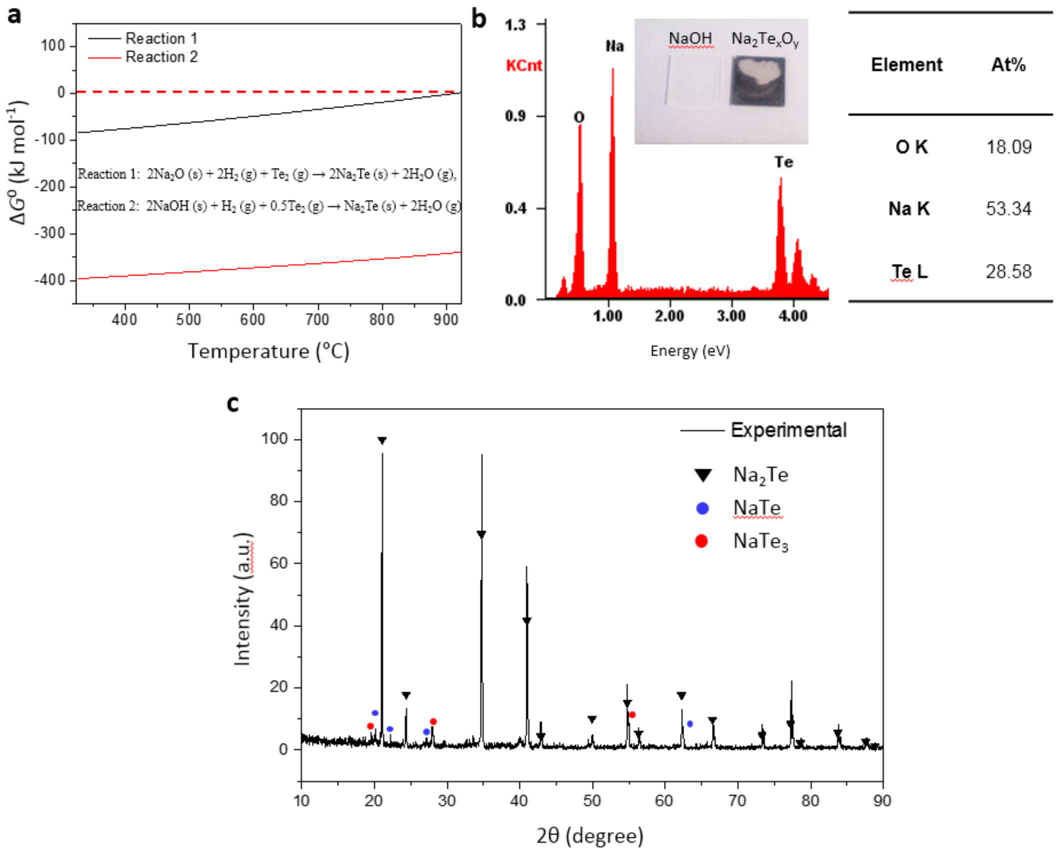

Supplementary Figure 2 | Telluriding NaOH for formation of sodium scooter (Na_2Te). a, Gibbs free energy calculation for the reactions of telluriding NaOH. **b,** Energy dispersive spectroscopy spectra of tellurized NaOH sample which reveals the ratio of around 2 to 1 between Na and Te atom. **c,** XRD spectrum of tellurized NaOH sample.

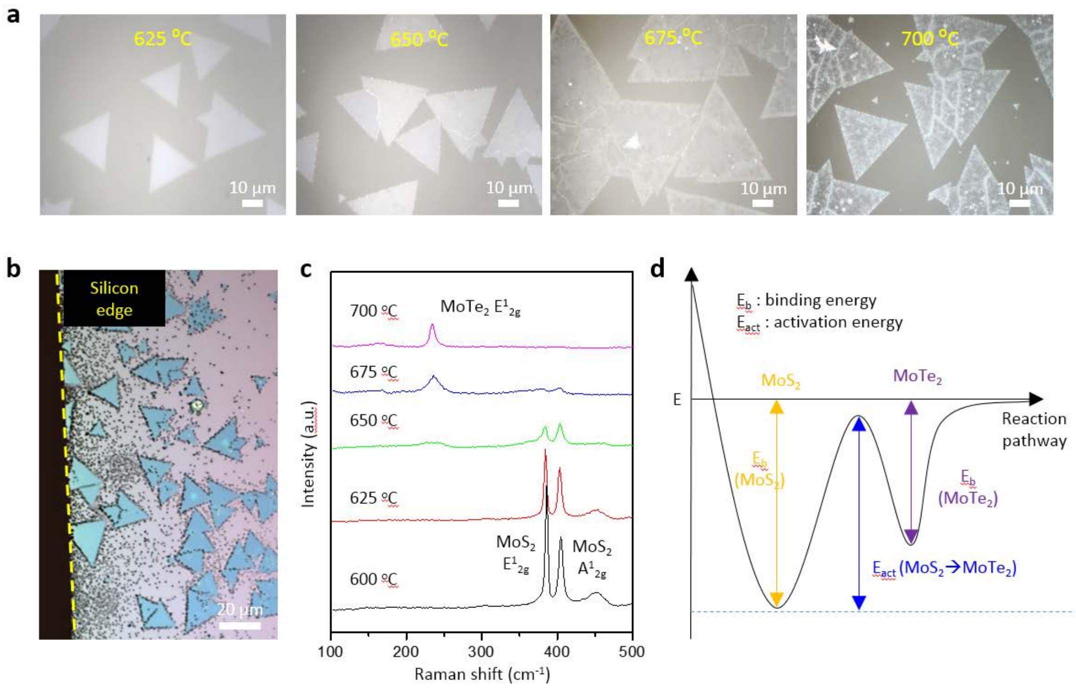

Supplementary Figure 3 | Tellurization of MoS_2 flakes without Na-scooter. a, Optical images of MoS_2 flakes which were tellurized at 625 °C, 650 °C, 675 °C, and 700 °C for 2h without a Na scooter. The triangular shape of the flakes was etched at temperatures over 650 °C. It should be noted that silicon telluride can be formed and deposited on MoS_2 flakes (Supplementary Figure 3b) which could lead to hindering the conversion of MoS_2 to $MoTe_2$ when a SiO_2/Si wafer is used as a substrate. To avoid this, we used a sapphire substrate especially for the high-temperature tellurization process (over 650 °C). **c,** The corresponding Raman spectra of the tellurized MoS_2 samples. Even though the E^1_{2g} mode for 2H-$MoTe_2$ appeared over 650 °C, the structure was broken at this temperature. **d,** Reaction pathway for the conversion from Mo–S to Mo–Te. The activation barrier height for converting MoS_2 to $MoTe_2$ is higher than the Mo–Te binding energy.

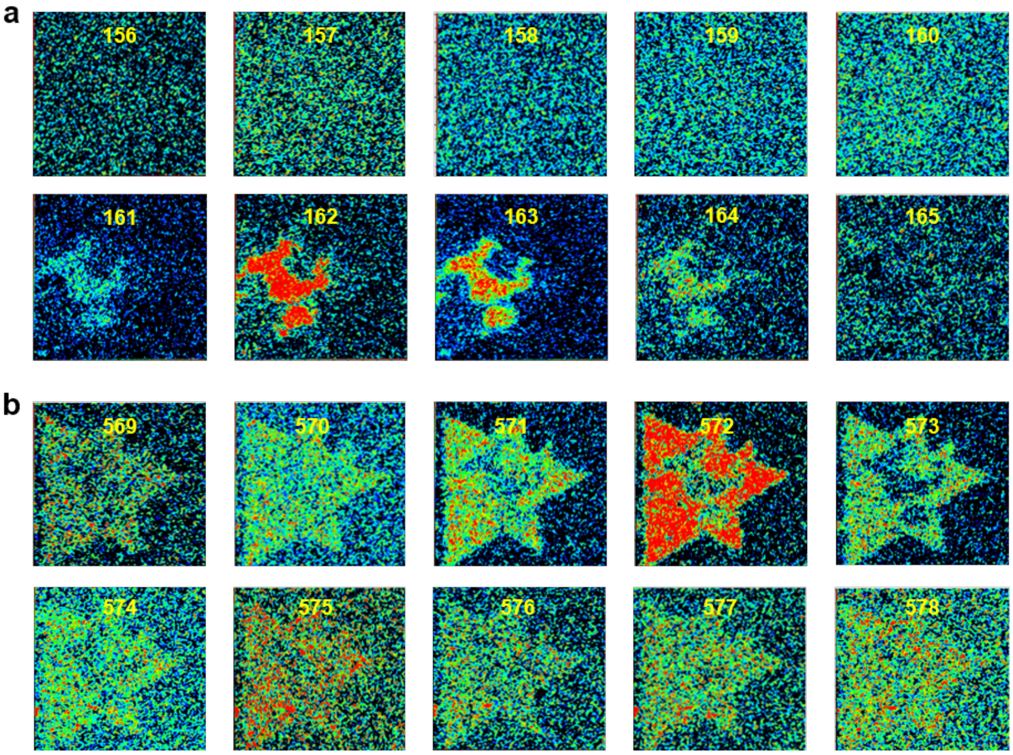

Supplementary Figure 4 | X-ray photoelectron spectroscopy (XPS) mapping images of 15-min tellurized MoS_xTe_{2-x} at different binding energies. a, XPS mapping images extracted from 156 eV to 165 eV for detecting the S $2p_{3/2}$ (162 eV) peak. **b,** XPS mapping images extracted from 569 eV to 578 eV for detecting the Te $3d_{5/2}$ (572 eV) peak.

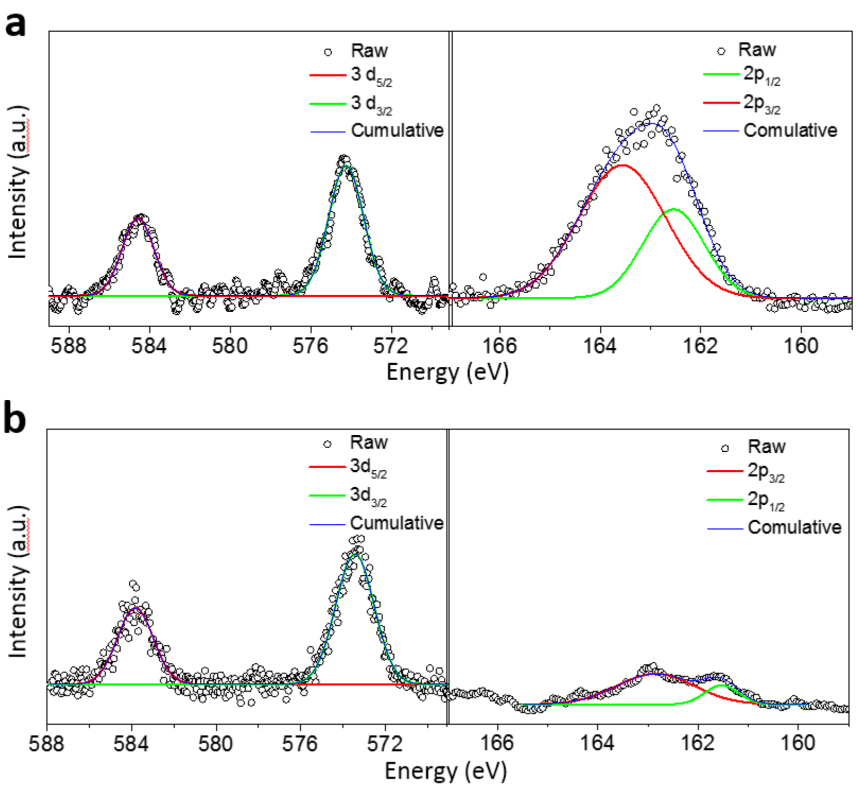

Supplementary Figure 5 | Representative XPS spectra in MoS_2 sample tellurized for 15 min. a, Representative XPS spectra in bright (inner) region. **b,** Representative XPS spectrum in dark (outer) region. The calculated S-to-Te ratios are 0.4 to 1.6 (inner region) and 0 to 2.0 (outer region), respectively. The red-shifted peaks for S $2p_{3/2}$ compared with the inner and outer regions indicate that tellurium alloying gives a p-doping effect to MoS_2 due to its shifting of the Fermi level.

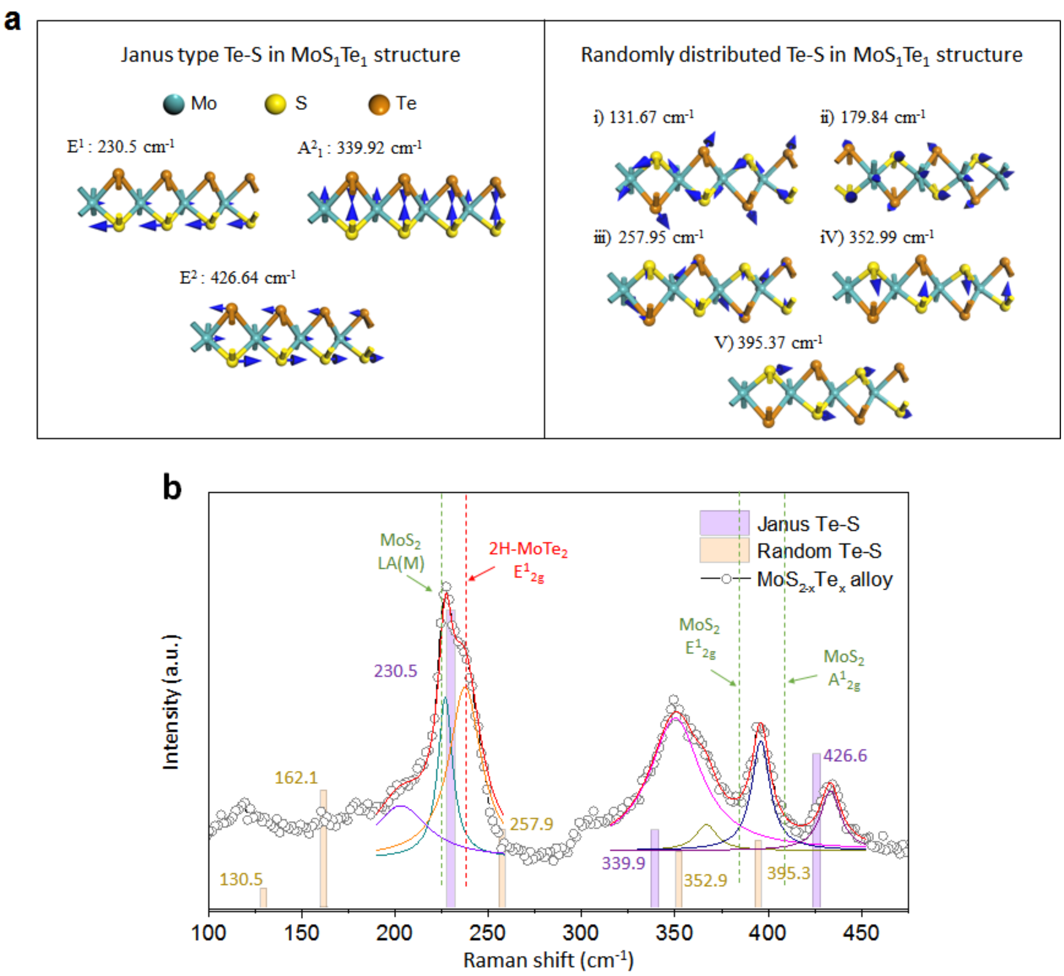

Supplementary Figure 6 | Calculated phonon modes for the Janus-phase and random Te-S distributed MoS_1Te_1 structure. a, Schematic for various phonon vibration modes of the Janus-phase and random Te-S distributed MoS_1Te_1 structure. **b,** The comparison between deconvoluted Raman spectrum of $MoS_{2-x}Te_x$ alloy and calculated Raman modes of Janus and Random Te-S distributed MoS_1Te_1 by the local density approximation.

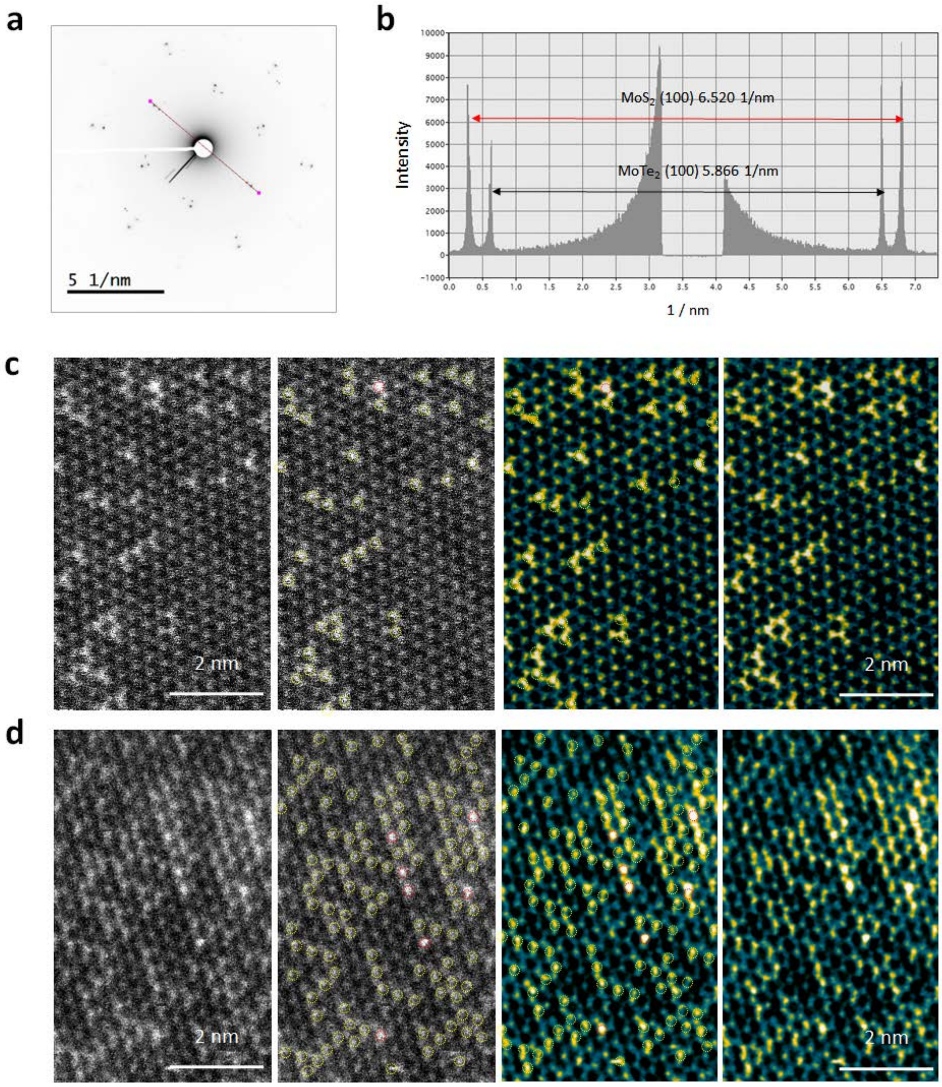

Supplementary Figure 7 | TEM analysis of MoS_xTe_{2-x} alloy. a, Electron diffraction pattern of the MoS_xTe_{2-x} alloy. Another six dots inside the diffraction patterns of 2H-MoS_2 were seen with the same orientation. **b,** Extracted lattice distances from the hexagonal diffraction patterns indicate that the inner and outer distances are 0.34 nm and 0.306 nm which are equivalent to $MoTe_2$ and MoS_2 phases, respectively. **c,d,** Raw and false-colored FFT filtered ADF-STEM images of region 1 and region 3 in Figure 3a. The two images on the left are the raw ADF-STEM image and the chalcogen site marked image (Te–S in the yellow circle and Te–Te in the red circle). After false-colored FFT filtering, Te substitution sites are clearly distinguished.

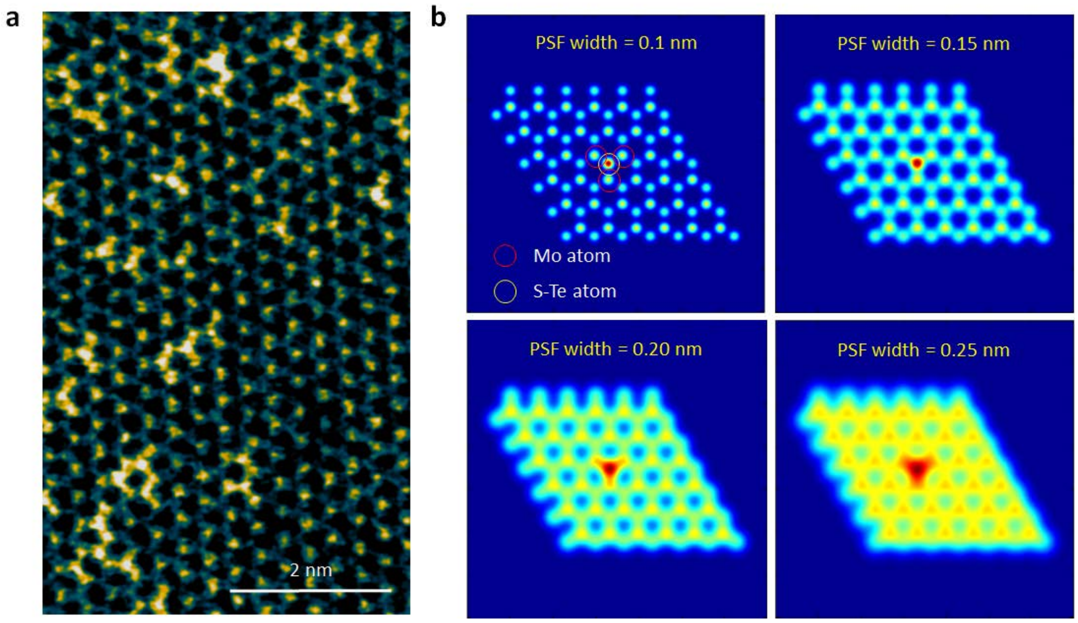

Supplementary Figure 8 | Effect of point spread function width on STEM simulation. a, FFT-filtered STEM image of $MoS_{2-x}Te_x$ alloy where adjacent Mo atoms to Te atoms show higher intensity than adjacent Mo atoms to S atoms. **b,** Simulated STEM images of 1Te atom substituted monolayer $MoS_{1.98}Te_{0.02}$ alloy with different point spread function (PSF) width. During FFT filtering, image can be modulated to increase the PSF width. To see the effect of PSF width on STEM image, we conducted STEM simulation depending on PSF width (0.1, 0.15, 0.2, 0.25 nm). It is clearly seen that the intensity of adjacent Mo atom to S-Te site is brighter in proportion to the PSF width.

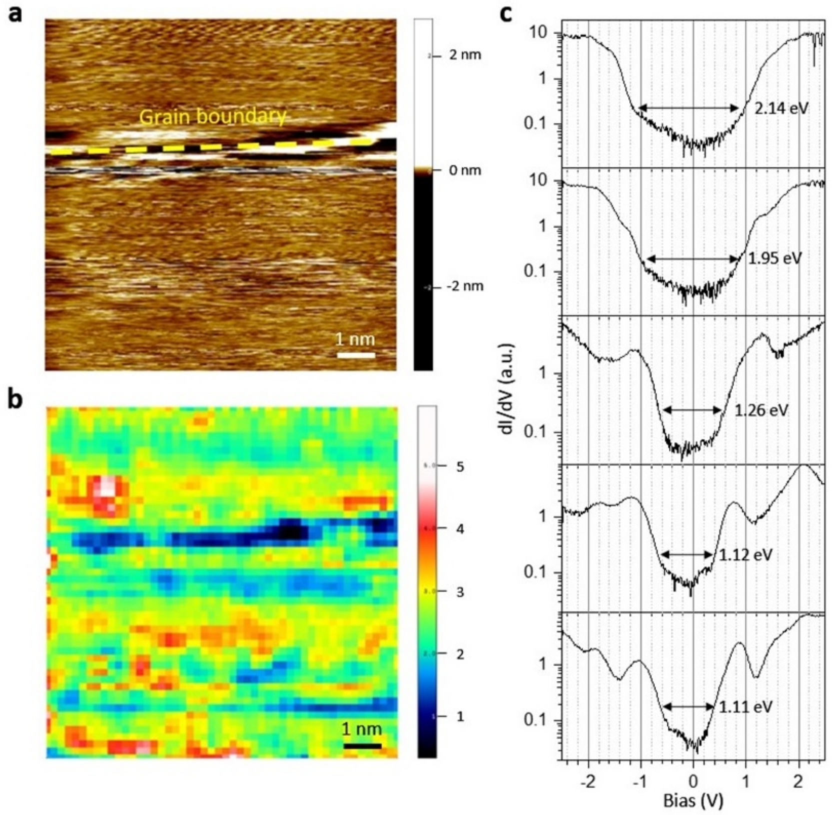

Supplementary Figure 9 | Various band gaps of tellurized MoS_2 by STS measurement. a, Surface morphology of tellurized MoS_2 on a HOPG substrate by scanning tunneling microscopy (STM) for STS mapping image. It shows a flat area with a grain boundary. The height fluctuation in the flat area has a range of ±0.1 nm without any significant height changes, which rules out the structural change by vacancy defects or the adsorption of atoms/molecules involving a height change of 0.3–0.6 nm in the surface morphology (sample bias = 2 V, tunneling current = 1 nA). **b,** STS map image at 1.47 V. Even for the similar surface morphology in the flat area of (a), the STS result shows a fluctuation of the electronic structures in tellurized MoS_2 from region to region. In addition, the grain boundary exhibits distinguishable dI/dV characteristics. **c,** Log-scale STS line profile results from region to region. The bandgap fluctuates over each flat region. The observed largest bandgap (2.14 eV) is similar to that of pristine MoS_2 on a HOPG substrate in previous report[1] and smallest observed bandgap (1.1 eV) is similar to reported bandgap of 2H-$MoTe_2$[2,3]. The bandgap tuning window by the tellurization of MoS_2 from 2.14 eV to 1.1 eV is nearly twice that of $MoS_{2-x}Se_x$ or $Mo_{1-x}W_xS_2$ alloys. For further information of bandgaps in $MoS_{2-x}Te_x$ alloy, results from DFT calculations based on Janus structure are provided for comparison of the bandgap with non-Janus case (See Supplementary Figure 11). Note that these phases discuss here are distinguished with defects such as grain boundaries and point defects (See also Supplementary Figure 10).

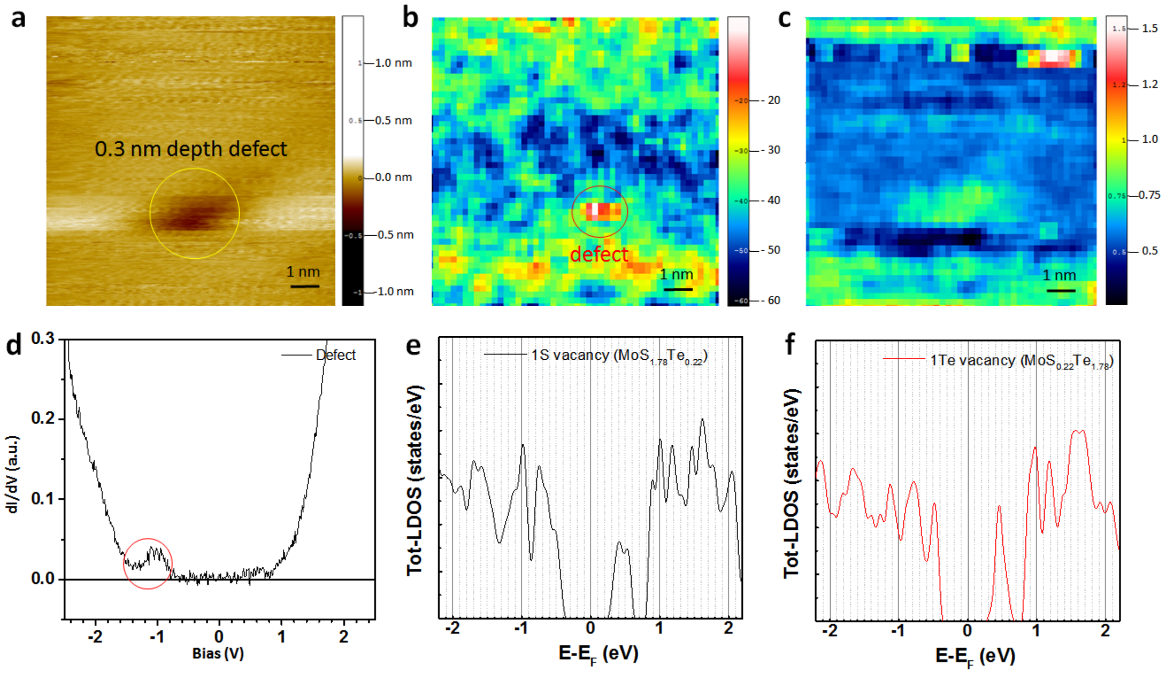

Supplementary Figure 10 | Defect features of tellurized MoS_2. a, Surface morphology by STM shows a defect site. The defect site exhibits a height difference of ~0.3 nm, which indicates a structural change with a loss of atoms from the intrinsic MoS_2 structure. Substitutional changes between S and Te atoms within the same topology of MoS_2 did not exhibit such a height variation at the angstrom scale. The STS map images at −1 V (b) and 2.5 V (c). Both images show defect-induced states. **d,** STS line profile at the defect site shows the defect state at −1 V. **e,f,** Band structure of $MoS_{0.22}Te_{1.78}$ with S and Te vacancies, respectively. Both S and Te vacancies generate a mid-gap band near the conduction band. In the STS results, the defect states were distinguished with surface morphological changes.

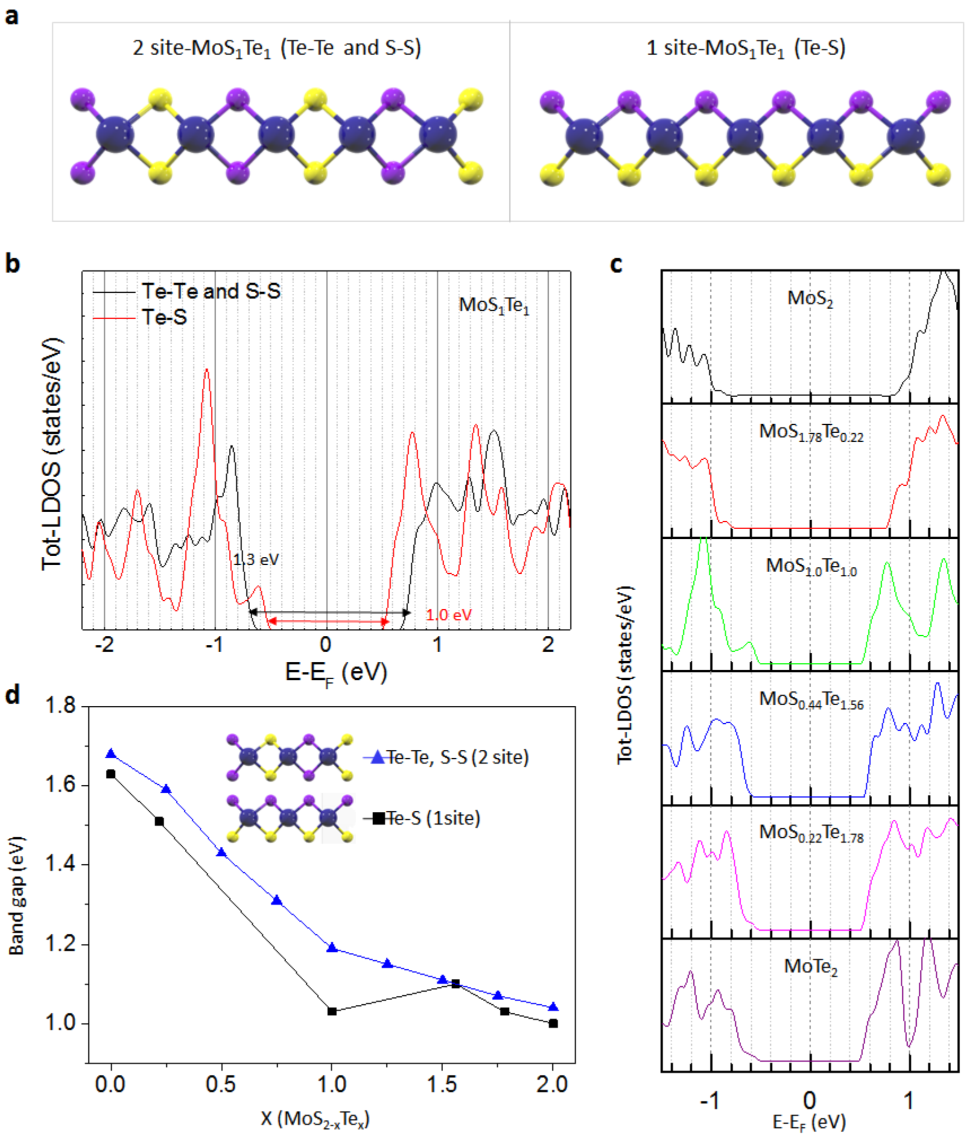

Supplementary Figure 11 | The band structure of the Janus-phase MoS_xTe_{2-x} alloy by density functional theory. a, Two types of atomic structure for the MoS_1Te_1 alloy. The alloy structure where S and Te atoms are located in the same vertical line is defined as the '2' site (top), whereas the other case is defined as the '1' site or Janus phase (bottom). **b,** The comparison of band structures between the 2 site and 1 site (Janus phase). It should be noted that the Janus phase (1.0 eV) has a smaller bandgap than the 2-site structure does (1.3 eV). **c,** The evolution of the band structure of the 1-site MoS_xTe_{2-x} alloy with different relative concentrations between the S and Te atoms. The gap decreases with increasing Te concentration. No bowing effect is revealed. **d,** Bandgap of the MoS_xTe_{2-x} alloy depending on the Te content. The blue triangular and black square regions are values from DFT calculations for 2-site (composed of Te–Te and S–S) [4] and 1-site (Te–S) phases. The bandgap is reduced in 1-site MoS_1Te_1 compared to that of the 2-site. Both cases show no bowing effect.

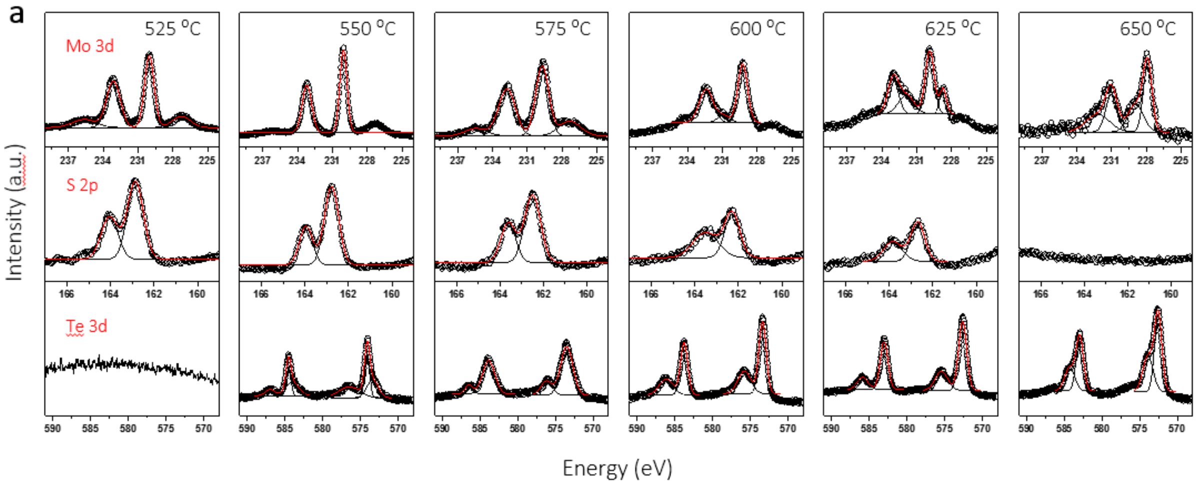

b

Tellurization Temp. (°C)	Mo 3d (eV) 3/2	Mo 3d (eV) 5/2	S 2p (eV) 1/2	S 2p (eV) 3/2	Te 3d (eV) 3/2	Te 3d (eV) 5/2	x ($MoS_{2-x}Te_x$)
**525**	233.11	230.03	162.85	164.04	-	-	0
**550**	233.20	230.06	162.77	163.94	584.53	574.15	0.33
**575**	232.76	229.72	163.61	162.49	583.87	573.51	0.54
**600**	232.40	229.25	163.49	162.27	583.78	573.43	0.87
**625**	231.90	228.73	163.83	162.68	583.05	572.67	1.03
**650**	231.00	227.88	-	-	582.95	572.55	2

Supplementary Figure 12 | XPS measurement for tellurized MoS_2 samples depending on the conversion temperature. a, Deconvoluted XPS spectra for Mo 3d, S 2p, and Te 3d of tellurized MoS_2 converted at different temperatures. From 600 °C, other peaks in Mo 3d were emergent. They are correlated with the Mo 3d value of 1T' and the 2H-phase $MoTe_2$ which are correlated with Raman analysis. **b,** The table for XPS peak positions (Mo 3d, S 2p, and Te 3d) and S-to-Te ratio of tellurized MoS_2. The Te content is proportional to the conversion temperature.

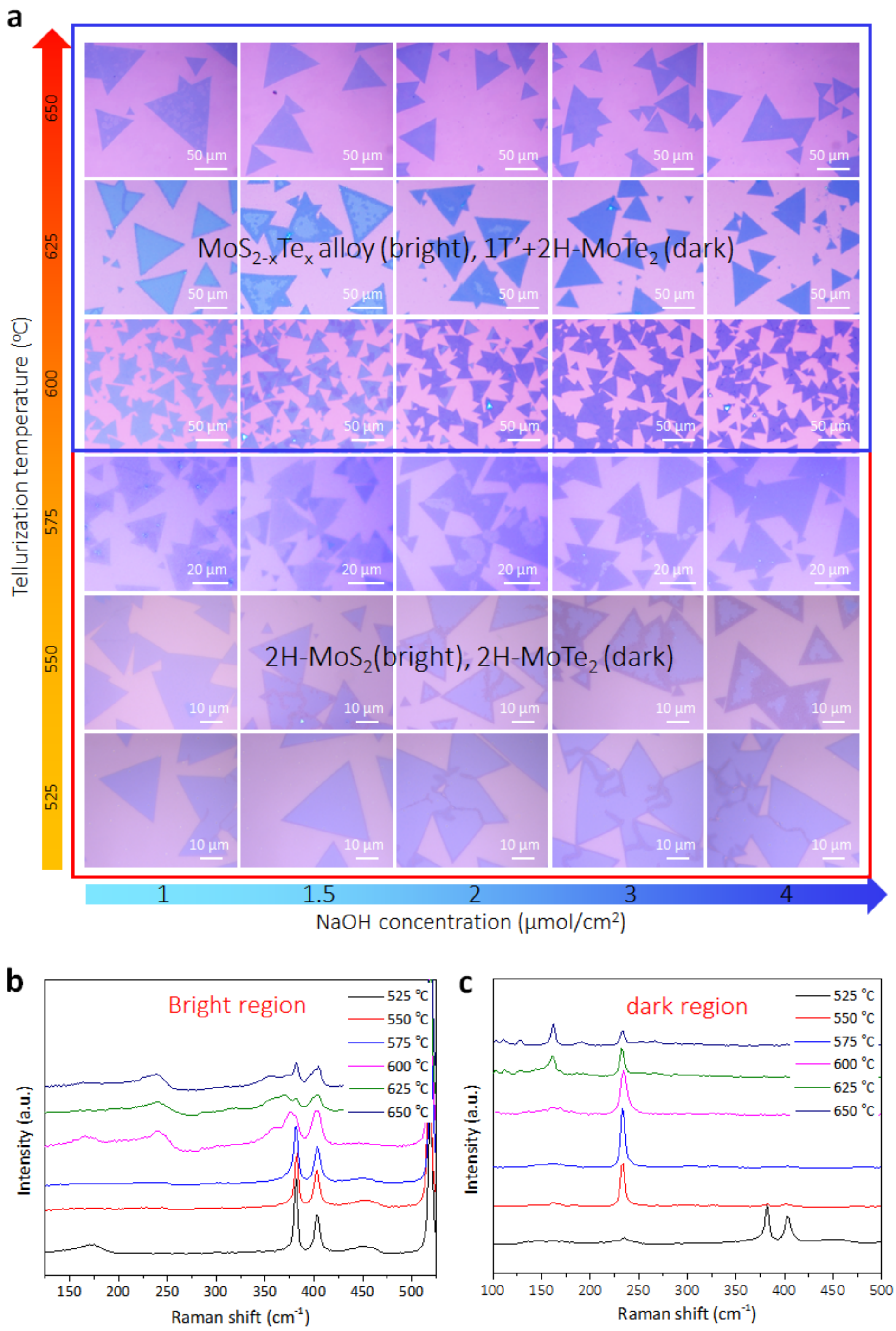

Supplementary Figure 13 | Temperature and NaOH concentration dependence on tellurization of MoS_2. a, The optical images of tellurized MoS_2 flakes depend on tellurization temperature and NaOH concentration. There are two distinct regions in terms of optical contrast (the edge and grain boundaries of flakes are dark and inner region of flakes are bright). **b-c,** Raman spectra of bright region (b) and dark region (c) for tellurized MoS_2 samples at various tellurization conditions. In temperature range from 525 °C to 575 °C, only MoS_2 peaks are

observed at bright regions because of the edge selective tellurization behavior. Over 600 °C, the basal surfaces of MoS_2 flakes are partially tellurized and converted to $MoS_{2-x}Te_x$ alloy. The dark regions in all tellurized samples show Raman spectra of fully converted $MoTe_2$. Only 2H-$MoTe_2$ phase are observed below 600 °C whereas mixed 1T' and 2H $MoTe_2$ phases are obtained over 600 °C. The small trace of MoS_2 peak in dark region in the bottom Raman spectrum (black color) is an artefact that due to too narrow area of 2H-$MoTe_2$ region, the laser spot size covered even MoS_2 region.

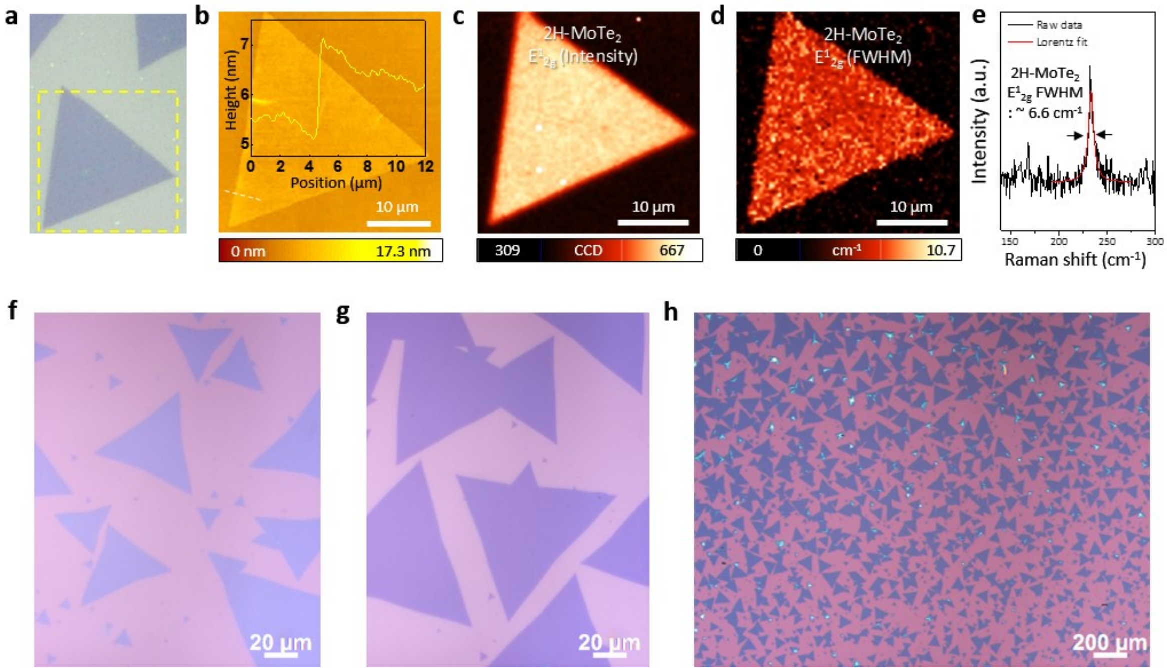

Supplementary Figure 14 | The uniformity and crystallinity of fully converted 2H-MoTe$_2$. a–e, Optical micrograph (a), AFM image (b), confocal Raman mapping images for the intensity of E^1_{2g} mode (c) and the FWHM of E^1_{2g} mode (d) of fully converted 2H-MoTe$_2$ and its representative Raman spectrum (e). The uniform optical contrast in (a) and AFM image (b) show no significant structural deformation in the μm range. Raman mapping images for the intensity (c) and FWHM (d) of E^1_{2g} mode for 2H-MoTe$_2$ indicate fully converted monolayer 2H-MoTe$_2$ flake with uniform crystallinity. The FWHM value for E^1_{2g} mode is around 6.6 cm^{-1} indicates that converted MoTe$_2$ has reasonable crystallinity compared to exfoliated one.

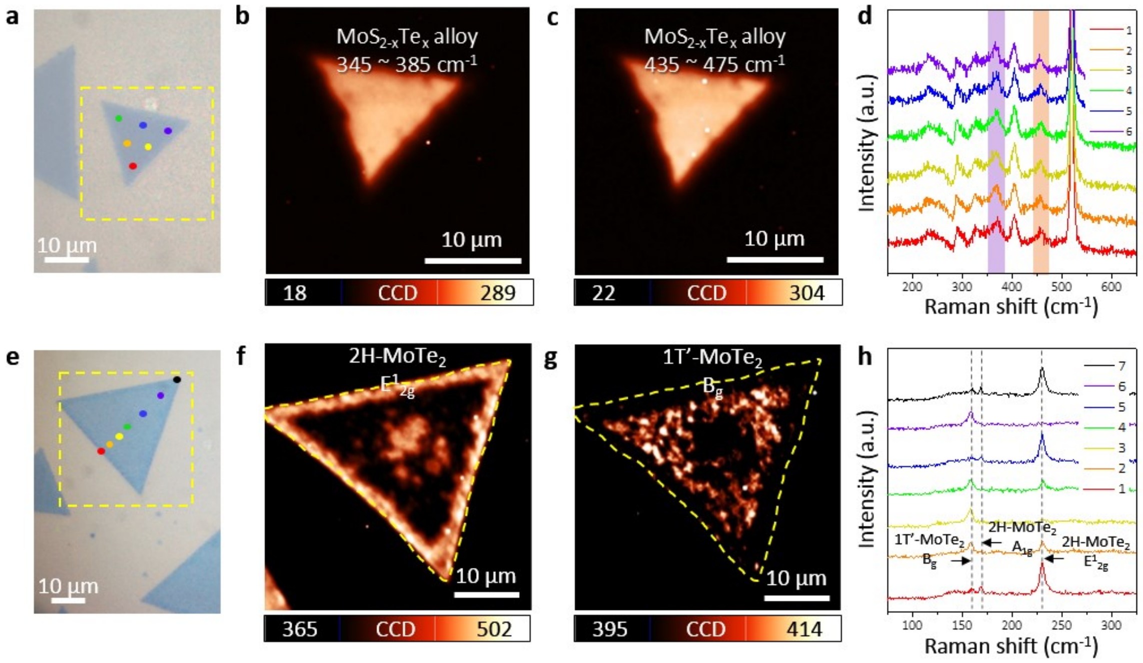

Supplementary Figure 15 | The sample uniformity of $MoS_{2-x}Te_x$ alloy and mixed 1T'-2H $MoTe_2$. a-d, optical micrograph (a) and Raman mapping images for $MoS_{2-x}Te_x$ alloy peaks (b and c) and its representative Raman spectra (d) marked in (a). The uniform contrast in Raman mapping images (b and c) indicate reasonable uniformity of $MoS_{2-x}Te_x$ alloy in a micrometer scale (sample grown with 625 ^{o}C and 1 μmol cm^{-2} for 30 min). **e-h,** optical micrograph (e), Raman mapping images for E^1_{2g} mode of 2H-$MoTe_2$ (f) and B_g mode of 1T'-$MoTe_2$ (g) and its representative Raman spectra (h) marked in (e). Each phase seems to be segregated to each other and the regions of 2H-$MoTe_2$ are located at the edge and center of flake, while 1T'-$MoTe_2$ exists in the middle of the flake. At the current stage, we are not sure why that kind of phenomenon took place. Single phase of 1T'-$MoTe_2$ is seen in regions marked by yellow and purple circle in (e). This indicates that there is possibility for fully converted single phase of 1T'-$MoTe_2$ in the inner area (g).

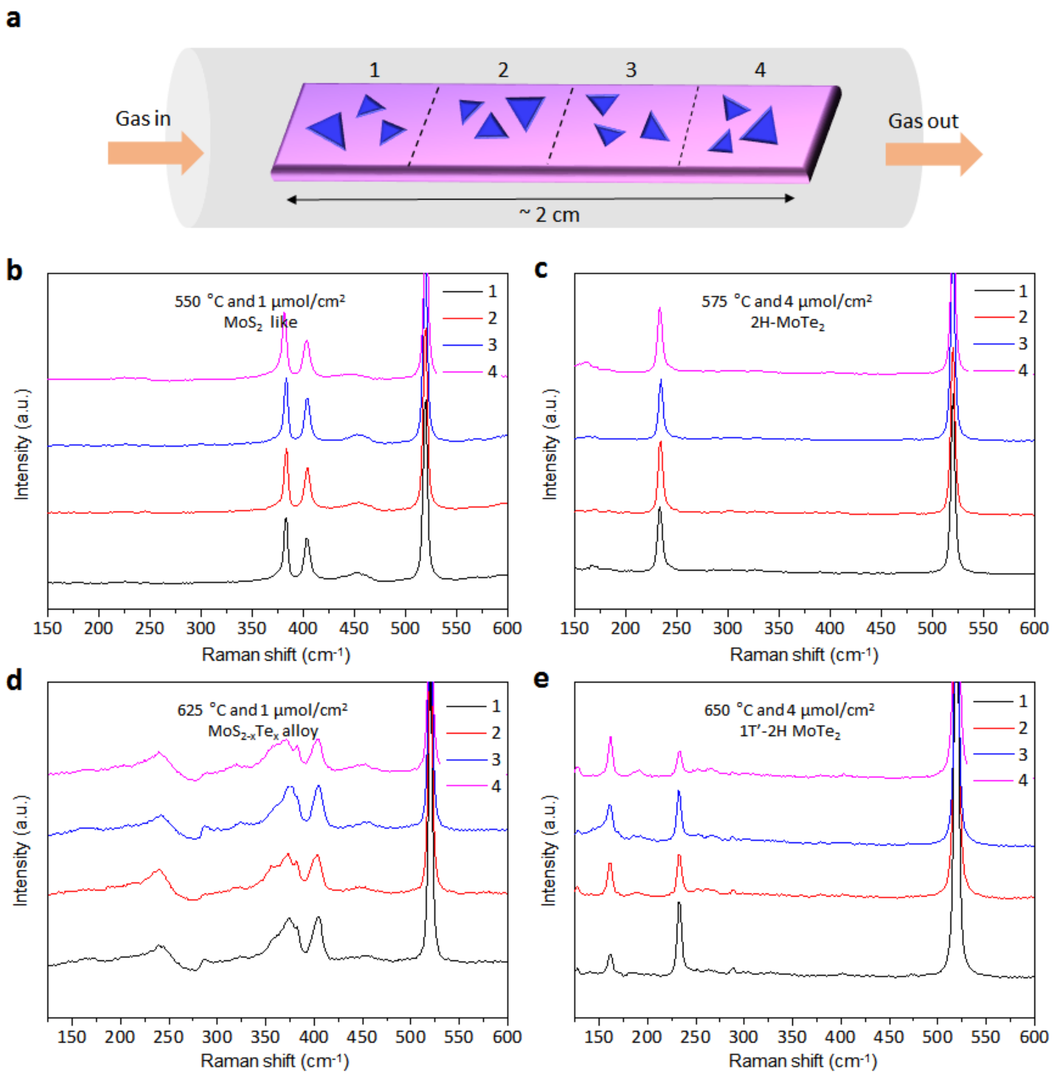

Supplementary Figure 16 | Sample uniformity of tellurized MoS_2 sample at different positions. To investigate uniformity with position, we measured Raman spectroscopy at four regions which are marked by the numbers in supplementary figure 16a for each sample. **b-d,** The position-dependent Raman spectra of tellurized MoS_2 at various telluriding conditions. We choose four representative conditions for MoS_2 like, 2H-$MoTe_2$, $MoS_{2-x}Te_x$ alloy and 1T'-2H $MoTe_2$, respectively. All the samples show similar Raman spectra to each other except the 1T'-2H $MoTe_2$ case (**e**) where the relative composition of 1T' phase to 2H phase is slightly different from each other.

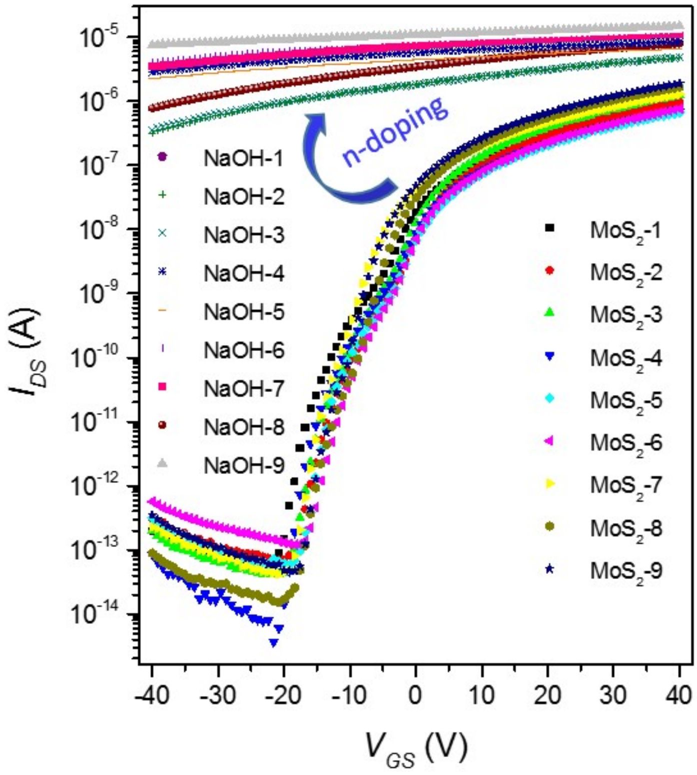

Supplementary Figure 17 | The effect of Na contamination on electrical transport behavior of MoS_2 FET. To confirm the effect of Na contamination to electrical transport of MoS_2 sample, we fabricated the nine number of MoS_2-FETs and measured electrical transport before and after NaOH doping. It is clear that Na-contamination gives n-type doping effect to MoS_2. Furthermore, there is a good reference which shows n-type doping effect of Na to MoS_2[5]. In this regard, our p-type doping behavior of tellurized MoS_2 does not originates from Na contamination but from Te substitution. .

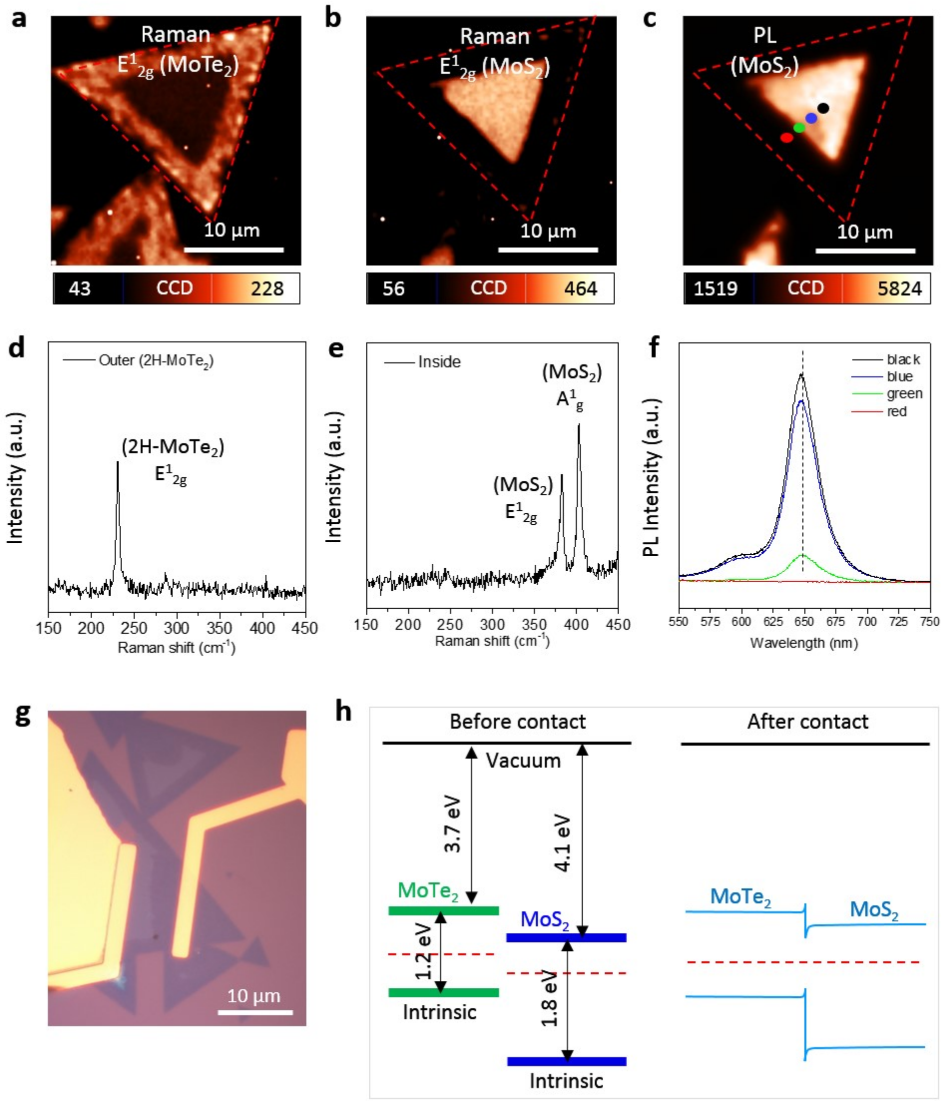

Supplementary Figure 18 | Lateral heterojunction between 2H-MoS_2 and 2H-$MoTe_2$. Raman mapping images (a, b) for the E^1_{2g} mode of 2H-MoS_2 ($MoTe_2$) and each representative spectrum (d, e). **c,f**, PL mapping image for MoS_2/$MoTe_2$ heterojunction and its representative PL spectra in marked regions in (c). PL is highly sensitive to doping. Inner part shows pristine MoS_2 PL spectrum. No distinguishable change in the peak position is observed across the junction, indicating that a sharp interface between MoS_2 and $MoTe_2$ is formed. **g,h,** Optical image of the MoS_2/$MoTe_2$ junction device (g) and its band alignment.

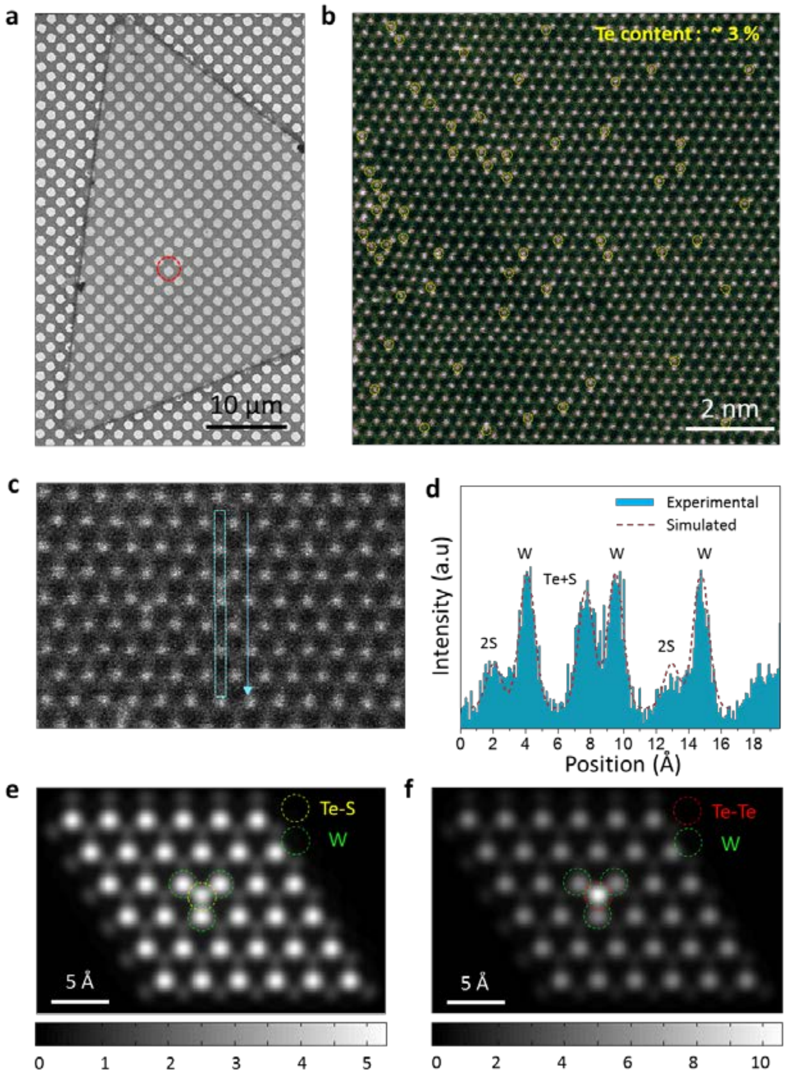

Supplementary Figure 19 | TEM analysis for inner part of tellurized WS_2 Sample. a, Low-magnification TEM image of the tellurized WS_2 flake on a 1.2-μm hole Cu grid. **b,** The ADF-STEM image for inner region (marked by red circle in supplementary figure 19a) of tellurized WS_2. Te content is approximately assumed to 3 percent by counting Te atoms. **c,d,** Representative ADF-STEM images of inner region and intensity profile in blue box in **c** respectively. Some bright spots were seen in chalcogen sites and it has lower intensity than W atom but higher than 2S atom indicating the existence of Te-S species. **e,f,** Simulated STEM image for Te-S site and Te-Te site in $WS_{2-x}Te_x$ alloy. Te-Te site has higher intensity than W site while Te-S site has similar intensity compared to W site.

Supplementary Tables

Supplementary Table 1 | Thermodynamic data table for tellurization of MoS_2.

Temperature (K)	G H_2(g) (kJ/mol) [6]	G H_2O(g) (kJ/mol) [6]	G MoS_2(s) (kJ/mol) [6]	G $MoTe_2$(s) (kJ/mol)[7]	G Na_2S(s) (kJ/mol) [6]	G Na_2Te(s) (kJ/mol) [6]	G Na_2O(s) (kJ/mol) [6]	G NaOH(l) (kJ/mol) [6]	G S_2(g) (kJ/mol) [6]	G Te_2(g) (kJ/mol) [6]
**600**	−−81.836	−359.23	−321.694	62.46	−433.742	−392.087	−471.851	−472.652	−12.304	0.63
**700**	−97.175	−380.838	−333.387	29.253	−449.987	−409.778	−485.456	−486.998	−34.817	−28.185
**800**	−112.937	−402.982	−346.155	−4.903	−467.503	−428.657	−500.326	−502.569	−63.848	−57.563
**900**	−129.07	−425.612	−359.879	−39.901	−486.147	−448.591	−516.336	−519.205	−90.336	−87.44
**1000**	−145.536	−448.687	−374.462	−75.662	−505.809	−469.479	−533.384	−536.779	−117.232	−117.769
**1100**	−162.305	−472.174	−389.829	−112.12	−526.412	−491.238	−551.516	−555.193	−144.496	−148.51
**1200**	−179.352	−496.049	−405.916	−149.222	−548.04	−513.115	−570.569	−574.363	−172.096	−179.633

Supplementary Table 2 | Cohesive energy and decomposition temperature of transition metal dichalcogenides.

Material	Cohesive energy (M–X) (eV)	Decomposition temp. (°C)
**MoS_2**	15.31[8]	~1090[9]
**$MoSe_2$**	13.70[8]	~980[9]
**$MoTe_2$**	12.01[8]	~700[9]
**WS_2**	17.28[8]	~1040[9]
**WSe_2**	15.45[8]	~ 930[9]
**WTe_2**	13.51[8]	~ 700[9]

Supplementary Note

Supplementary Note 1 | Na-scooter (Na_2Te) formation from NaOH.

Sodium metal reacts with tellurium during the tellurization process and forms a Na-scooter (Na_2Te). The NaOH can be thermally reduced to Na_2O by desorbing H_2O gas during a dry process. Therefore, we assume that the starting material could be NaOH or Na_2O. The expected reactions for forming Na_2Te in the presence of tellurium and hydrogen gas are written below.

Reaction 1: $2Na_2O\ (s) + 2H_2\ (g) + Te_2\ (g) \rightarrow 2Na_2Te\ (s) + 2H_2O\ (g)$,

Reaction 2: $2NaOH\ (s) + H_2\ (g) + 0.5Te_2\ (g) \rightarrow Na_2Te\ (s) + 2H_2O\ (g)$

To anticipate its favorability of reaction, we calculated the Gibbs free energy for the mentioned reactions in the temperature range of 323 °C to 923 °C (Supplementary Figure 2a)**.** Both reactions show negative values, indicating that Na_2Te formation is thermodynamically favorable from a NaOH precursor. Reaction 2 is regarded as more active than reaction 1 in terms of the Gibbs free energy.

To confirm Na_2Te formation, we conducted energy dispersive spectrometry (EDS) measurements for the NaOH-coated substrate after tellurization (Supplementary Figure 2b). The dark gray color and the presence of Na and Te peaks in the EDS measurements indicate that sodium telluride is formed. The atomic ratio of Na to Te is around 1.9, implying the likely formed Na_2Te complex. The detected O peak in the EDS measurement results from oxidized Na_2Te because it is very sensitive to air during sample transfer for measurement. X-ray diffraction (XRD) was used for further confirming the formation of Na_2Te (Supplementary Figure 2c). We used the sealed sample holder to avoid oxidation of tellurized NaOH sample. The XRD peaks from tellurized NaOH (experimental) are well matched with Na_2Te with very small feature of NaTe and $NaTe_3$, indicating that NaOH are mostly converted to Na_2Te during tellurization process.

Supplementary References

1. Shin, B.G. *et al.* Indirect bandgap puddles in monolayer MoS_2 by substrate-induced local strain. *Adv. Mater.* **28,** 9378-9384 (2016).
2. Lezama, I.G. *et al.* Surface transport and band gap structure of exfoliated 2H-$MoTe_2$ crystals. *2D Mater.* **1,** 021002 (2014).
3. Yu, Y. *et al.* Molecular beam epitaxy growth of atomically ultrathin $MoTe_2$ lateral heterophase homojunctions on graphene substrates. *Carbon* **115,** 526-531 (2017).
4. Rajbanshi, B., Sarkar, S. & Sarkar, P. The electronic and optical properties of $MoS_{2(1-x)}Se_{2x}$ and $MoS_{2(1-x)}Te_{2x}$ monolayers. *Phys. Chem. Chem. Phys.* **17,** 26166-26174 (2015).
5. Komesu, T. *et al.* A. Occupied and unoccupied electronic structure of Na doped MoS_2 (0001). *Appl. Phys. Lett.* **105**, 241602 (2014).
6. Barin, I. *Thermochemical Data of Pure Substances*. (Wiley-VCH, Wiley, 1997).
7. Mallika, C. & Sreedharan, O. Standard molar Gibbs energy of formation of $MoTe_2$ from emf measurements. *The Journal of Chemical Thermodynamics* **20**, 769-775 (1988).
8. Kang, J., Tongay, S., Zhou, J., Li, J. & Wu, J. Band offsets and heterostructures of two-dimensional semiconductors. *Appl. Phys. Lett.* **102**, 012111 (2013).
9. Brainard, W. A. *The thermal stability and friction of the disulfides, diselenides, and ditellurides of molybdenum and tungsten in vacuum*. (National Aeronautics and Space Administration, 1968).
